# Cross-Sectional associations between inner setting determinants of self-efficacy and intent to deliver a healthy eating and activity curriculum embedded in a community setting

**DOI:** 10.1186/s12966-025-01736-5

**Published:** 2025-04-10

**Authors:** Rachel G. Tabak, Cynthia D. Schwarz, Allison Kemner, Debra Haire-Joshu

**Affiliations:** 1 School of Public Health, Washington University in St. Louis, St. Louis, MO 63130 USA; 2 School of Public Health, Washington University in St. Louis, St. Louis, MO USA; 3 Parents as Teachers National Center, Creve Coeur, MO USA; 4 School of Public Health, Washington University in St. Louis, St. Louis, MO USA

**Keywords:** Healthy eating and activity, Implementation, Home visiting

## Abstract

**Background:**

Healthy Eating and Active Living Taught at Home (HEALTH) embeds healthy eating and activity content within Parents as Teachers (PAT), a national home visiting program. HEALTH is evidence based to prevent weight gain among mothers of young children. This secondary analysis aims to understand the factors associated with intention and self-efficacy to deliver HEALTH among parent educators (home vising providers).

**Methods:**

This is a cross-sectional, secondary analysis of data from a trial evaluating the effectiveness of HEALTH when delivered by parent educators as part of usual practice. Parent educators completed surveys following training in the HEALTH intervention; demographic characteristics (including self-reported body mass index) were collected in a baseline survey (pre-training). Surveys were based on two implementation science frameworks: Consolidated Framework for Implementation Research (CFIR, implementation context) and Reach, Effectiveness, Adoption, Implementation, and Maintenance (RE-AIM, implementation outcomes). Associations between intent to deliver HEALTH (intent) and self-efficacy (SE) to deliver HEALTH, implementation context constructs and demographic characteristics were explored using Pearson correlations (continuous variables) and t-tests (binary variable). Relationships were considered significant if the p-value was < 0.05.

**Results:**

Among the 149 parent educators who completed the survey, just over half identified as white/non-Hispanic (53%), while just over a third identified as Hispanic. Participants reported having worked at their site for a mean of 4.7 years (standard deviation, SD = 5.85), and the mean body mass index was 30.43 kg/m^2^ (SD = 7.35). There was a significant correlation between intent and SE, *r* = 0.46 (< 0.0001). Most demographic characteristics (e.g., body mass index, age) were not significantly correlated with either variable, however, intent and SE were both significantly lower among white non-Hispanic parent educators than among those identifying as another race/ethnicity. Several other implementation context constructs such as evidence strength and quality, mission alignment, appeal, openness, and relative advantage were positively correlated with both intent and SE; complexity was negatively correlated.

**Conclusions:**

When implementing healthy eating and activity content within community settings, it is important to consider what factors may be related to provider intent and provider self-efficacy to deliver the content. Specifically, mission alignment, complexity, evidence strength and quality, and relative advantage may be important.

**Trial registration:**

: NCT03758638 (https://clinicaltrials.gov/study/NCT03758638), registered Nov 29, 2018.

## Background

Community providers have important skills in interacting with people, particularly those who may not be connected with clinical care. Additional training in healthy eating and activity content among those working in community settings can leverage the skills they already have in reach and engagement. Given community organizations are not designed to deliver health promotion content, there is a need to explore factors (e.g., intention to deliver interventions and provider self-efficacy regarding content) associated with implementation outcomes [1, 2]. Potential factors to consider include contextual determinants of implementation, such as characteristics of the providers themselves (e.g., age, race/ethnic identity) and aspects of the organization where they work, such as how the healthy eating and activity content fits with the organization’s mission. Assessing these factors allows exploration of whether they are related to implementation outcomes such as adoption to better inform strategies to support implementation [3]. 

Researchers have looked at the relationships between contextual determinants and implementation outcomes related to implementation in community settings such as faith communities and early care and education organizations [4–6]. These studies have identified numerous contextual determinants such as barriers and facilitators to implementation, adoption, and sustainability [7]. Examples of contextual factors that have been evaluated include: alignment with workflow/ routines, adequate time to deliver intervention content, priorities, financial factors (e.g., reimbursement, cost), values regarding health promotion, trust and rapport between implementers and those they serve, awareness, knowledge, skills, attitudes, confidence, and beliefs about the intervention.

It is important to expand the explorations of associations between these outcomes within community settings in general, as this research has been limited to date [2]. Given the diversity of community-based settings, it is important to see if findings from faith-based and early care and education settings are similar in home-visiting organizations. Home visiting organizations operate differently than faith-based and early care and education settings as providers go out of the organization to participant homes; home-visiting programs also serve the whole family, rather than just the children in their care as in early care and education settings [8]. Further, anecdotally, providers in ongoing studies have expressed differences in their comfort with implementation based on their own weight status. While comfort discussing weight has been explored among other providers (e.g., school nurses) [9, 10], literature exploring relationships between community-based providers weight status and adoption and implementation of an intervention focused on weight-related behaviors was not available. Further, while some anecdotal reports suggest that providers with higher BMIs appreciate the information and are excited to share the information with those they serve, while other reports providers with high BMI are hesitant to discuss behaviors related to weight given their own weight status. Implementation within home visiting is important as they are experts in engaging with parents and parental behavior and home environment are important in shaping behavior and weight outcomes in young children [11–14]. 

Parents as Teachers (PAT) is an international, evidence-based home visiting program delivered through organizations in all 50 states and several other countries. PAT empowers parents as their child’s first and most influential teacher, encouraging positive parent-child communication and increasing parents’ knowledge of child development and ways to stimulate social and physical development. HEALTH (Healthy Eating and Active Living Taught at Home) embeds healthy eating and activity content within PAT’s usual practice and is evidence-based to reduce weight gain among mothers of young children [15] and to improve to the home environment to support healthy eating among parents and children [16]. This secondary analysis aims to understand the contextual determinants that are associated with intention to deliver the HEALTH intervention and the self-efficacy of parent educators (home visiting program providers) to deliver HEALTH (implementation outcomes) within home visiting [17]. 

## Methods

This is a cross-sectional, secondary analysis of data from a cluster randomized trial evaluating the effectiveness of HEALTH when delivered by parent educators as part of usual home visiting practice. Additional details of the trial are published elsewhere; [17] briefly, it is a pragmatic cluster randomized controlled trial to evaluate dissemination and implementation of the evidence-based HEALTH intervention on weight among mothers with overweight and obesity across the United States. Three levels (mother, parent educator, PAT site) are considered; the current analysis focuses on the parent educator level. Parent educators are hired from the communities they serve, are trained and certified on the PAT Foundational curriculum and model implementation (usual care model), meet with parents and caregivers to coach positive parenting skills and interactions; conduct regular child screenings; connect families to services and resources as appropriate, and stay with families over time [18]. All parent educators working at sites (clusters) randomized to the HEALTH arm were eligible to participate in the study and the HEALTH training (training is described below). Those interested in participating completed a verbal consent process with a study team member before completing data collection surveys.

### Parent educator training

From January 2018 through June 2024, parent educators at PAT sites randomized to the intervention arm were trained in the HEALTH curriculum. The curriculum trainings took place over 10 hours (split between 2 days based on the trainees’ schedules). The trainings included education about healthy eating and activity, review of curriculum materials, role-playing, and discussion.

### Measures

Surveys were selected to measure the constructs in two implementation science frameworks: Consolidated Framework for Implementation Research (CFIR, implementation context) [19–21] and Reach, Effectiveness, Adoption, Implementation, and Maintenance (RE-AIM, implementation outcomes) [22, 23]. Survey measures (e.g., Measure of Innovation-Specific Implementation Intentions (MISII); Evidence-Based Practice Attitude Scale (EBPAS)) were identified from the literature [24–30] and had been previously pilot-tested among home visiting staff [31]. The CFIR was originally developed to guide understanding of the context for implementation including the providers delivering the intervention (in this case parent educators delivering HEALTH), factor related to the intervention (in this case HEALTH), and characteristics of the organization delivering the intervention (in this case PAT sites). In 2022, the CFIR framework was updated and an outcomes addendum was suggested, which included antecedent outcomes (potential predictors of adoption or implementation) [20, 21]. In accordance with this update, a careful comparison between the original framework and updated framework led to adaptations to how CFIR was operationalized in the current study. Constructs were re-organized and re-defined as suggested by the 2022 CFIR update and Outcomes Addendum; some constructs were also moved to the Outcomes Addendum. The RE-AIM framework guides the assessment of implementation outcomes. Table 1 shows how the survey measures used in the study map to CFIR and RE-AIM. There were several constructs of interest across the CFIR individuals/innovation delivers, inner setting, and innovations domains. One survey measure was used to assess each of these constructs. Within RE-AIM, which has broader domains without underlying constructs, multiple survey measures were used to assess the domain, including one survey measure used to assess one of the two primary outcomes for the current study, implementation intentions (RE-AIM adoption). The other primary outcome in the current analysis parent educator self-efficacy (CFIR individuals domain) [31]. These frameworks are commonly employed in studies testing the implementation of healthy eating and activity interventions within community settings [1, 4–7, 32, 33]. 

### Data collection

Parent educators completed surveys following training in the HEALTH intervention; demographic characteristics (including self-reported height and weight, used to calculate body mass index) were collected in a baseline survey (pre-training). Data collection ranged from February 2019 to May 2023.

### Analysis

Scales were calculated as the mean of the included items (Table 1) as recommended by previous use of the surveys. All variables except race/ethnicity were treated as continuous variables; race/ethnicity was categorized as (White, Black/African American, non-Hispanic; and Hispanic). For parent educators with missing data for any item in a scale, that entire scale was treated as missing, and for bivariate analyses, any parent educator with a missing values for either of the two variables was dropped from that analysis. Thus, the sample size for each univariate variable and bivariate test is presented. Associations between intent to deliver HEALTH (intent) and other constructs from CFIR and RE-AIM as well as demographic characteristics were explored using Pearson correlations (continuous variables) and t-tests (binary variable). This analysis was repeated for self-efficacy (SE) to deliver HEALTH. A formal power calculation was not used to determine the sample size for this analysis. Relationships were considered significant if the p value was less than 0.05.

## Results

Of the 169 participants who completed the baseline survey 149 completed the survey post training in the HEALTH curriculum. The number of participants for each survey measure is shown, as participants could skip any items the preferred not to answer (**Table 1**). Just over half of participants identified as white/non-Hispanic (53%), while just over a third identified as Hispanic. Participants reported having worked at their site for a mean of 4.7 years (standard deviation, SD = 5.85) and had been at their job a similar amount of time 4.3 years (SD = 5.56). The mean body mass index was 30.43 kg/m^2^ (SD = 7.35).

**Table 1:** Descriptive statistics of CFIR implementation determinant and RE-AIM implementation outcomes survey measures

CFIR/RE-AIM Domain/ Construct	Sample item	# of items^*^	*N*	Mean	Std Dev	
**Individuals Domain: Innovation Deliverers**						
**Knowledge and Beliefs About HEALTH**(30)^a^	I don’t know what a Healthy Lifestyle curriculum is.^bc^	4	145	2.90	0.47	
**PE Characteristics: Self-Efficacy**(26, 28)^d^	I am confident that I can implement HEALTH as prescribed at our site.	4	140	6.40	0.73	
**PE Characteristics: Demographics**						
Self reported body mass index			135	30.43	7.35	
Age (years)			146	39.52	12.34	
Years working at site			146	4.71	5.85	
Years working at current position			145	4.26	5.56	
Race/Ethnicity			n	%		
White, non-Hispanic			70	53.03		
Black/African American, non-Hispanic			17	12.88		
Hispanic			45	34.09		
**Inner Setting Domain**						
Mission Alignment(26, 28)^d^	HEALTH fits well with the mission or overall goals of our site.	4	142	6.34	1.00	
**CFIR Innovation domain**						
Innovation Complexity^a^	The HEALTH curriculum would require complex teaching strategies.^b^	4	146	3.66	0.58	
Evidence Strength and Quality^d^	The proposed implementation of HEALTH should be effective, based on current scientific knowledge.	3	121	4.37	0.67	
Innovation Relative Advantage^a^	In general, the HEALTH curriculum would be more effective in helping women avoid gaining weight than our current curriculum practices.	4	144	3.45	0.49	
**RE-AIM Adoption**			N^*^	Mean	Std Dev	
Implementation Intentions^e^	I will use all aspects of HEALTH with families	3	145	3.31	0.72	
Appeal(24)^e^	If you received training in a curriculum that was new to you, how likely would you be to adopt it if it was intuitively appealing?	4	146	3.19	0.63	
Requirement (24)^e^	If you received training in a curriculum that was new to you, how likely would you be to adopt it if it was required by your supervisor?	4	144	2.59	1.14	
Openness(24)^e^	I like to use a new curriculum to help families.	3	145	3.39	0.61	
**RE-AIM Implementation**						
Divergence(24)^e^	Experience working in the field is more important than using a manualized curriculum.^b^	4	142	2.97	0.86	

^*^#, number of items, N, mean, and SD are for the scales, sample items are listed for reference

^a^Response options: Strongly disagree; Somewhat disagree; Somewhat agree; Strongly agree

^b^Questions were reversed coded

^c^Question wasn’t reverse coded in the reference paper. However, we reversed coded based on common sense

^d^Response options: Strongly disagree; Disagree; Somewhat disagree; Neither agree or disagree; Somewhat agree; Agree; Strongly agree; Don’t know

^e^Not at all; To a slight extent; To a moderate extent; To a great extent; To a very great extent

Abbreviations: CFIR, Consolidated Framework for Implementation Research; RE-AIM, Reach, Effectiveness, Adoption,

Implementation, and Maintenance; PE, parent educators; N, sample size; SD, standard deviation

There was a significant correlation between the two outcome variables of implementation intentions and self-efficacy (*r* = 0.46, *p* < 0.0001, *n* = 139). Most demographic characteristics (i.e., body mass index, age, or years at job/site) were not significantly correlated with either outcome (Table 2). However, implementation intentions were lower among parent educators who identified as white non-Hispanic (mean = 3.07, SD = 0.78) than among those identifying as another race/ethnicity (mean = 3.52, SD = 0.62); this was also the case for self-efficacy (mean among parent educators identifying as white non-Hispanic = 6.27, SD = 0.87; mean among parent educators not identifying as white non-Hispanic = 6.58, SD = 0.46). These differences were both statistically significant (implementation intentions: -t = 3.72, *p* = 0.0003; self-efficacy: t=-2.61, *p* = 0.01).

Measures for several other implementation context constructs within the innovation domain in CFIR were positively correlated with self-efficacy and implementation intentions. The relationship was positive with evidence strength and quality (self-efficacy: *r* = 0.41, *p* < 0.0001; intent: *r* = 0.31, *p* = 0.0007) and relative advantage (self-efficacy: *r* = 0.27, *p* = 0.0011; intent: *r* = 0.26, *p* = 0.0017). The items measuring complexity were reverse scored, so the positive correlation indicates self-efficacy (*r* = 0.37, *p* < 0.0001) and implementation intentions (*r* = 0.22, *p* = 0.0074) increase as complexity decreases. Awareness of the intervention, which CFIR describes as a characteristic of the individual delivering the intervention, was only significantly correlated with self-efficacy (*r* = 0.23, *p* = 0.0069), not with implementation intentions (*r* = 0.13, *p* = 0.11). Mission alignment, a construct in the CFIR inner setting domain, was positively correlated with both self-efficacy (*r* = 0.48, *p* < 0.0001) and implementation intentions (*r* = 0.29, *p* = 0.0005). Adoption and implementation, two implementation outcomes suggested by RE-AIM, were each correlated with implementation intentions and self-efficacy, but only by one metric for each outcome (Table 2).

**Table 2:** Bivariate relationships between Self-Efficacy and intent to deliver HEALTH and survey measures assessing constructs from CFIR and RE-AIM:

	Pearson Correlation Coefficients (*r*|*p*)^*^			
	Intent	n	Self-Efficacy at Post	n (SE)
CFIR Constructs				
Self-reported body mass index	-0.04 (0.68)	134	0.04 (0.63)	128
Age (years)	-0.09 (0.29)	144	0.00 (0.95)	139
Years working at site	-0.02 (0.83)	144	0.00 (0.97)	139
Years working at current position	-0.05 (0.55)	143	0.08 (0.38)	138
Knowledge and Beliefs About HEALTH	0.13 (0.11)	144	0.23 (0.0069)	140
Mission Alignment	0.29 (0.0005)	141	0.48 (< 0.0001)	137
Innovation Complexity	0.22 (0.0074)	145	0.37 (< 0.0001)	140
Evidence Strength and Quality	0.31 (0.0007)	120	0.41 (< 0.0001)	117
Innovation Relative Advantage	0.26 (0.0017)	143	0.27 (0.0011)	138
RE-AIM Domains				
Appeal	0.45 (< 0.0001)	144	0.36 (< 0.0001)	139
Requirement	-0.06 (0.44)	142	-0.10 (0.25)	137
Openness	0.69 (< 0.0001)	144	0.46 (< 0.0001)	139
Divergence	-0.04 (0.67)	141	-0.04 (0.63)	136

^*^all analyses are unadjusted

Abbreviations: CFIR, Consolidated Framework for Implementation Research; RE-AIM, Reach, Effectiveness, Adoption, Implementation, and Maintenance; PE, parent educators; N, sample size; SD, standard deviation

## Discussion

This study explored contextual determinants that predict intention to deliver the HEALTH intervention and the self-efficacy of parent educators to deliver HEALTH. Organizing the study based on the CFIR and RE-AIM frameworks allows the findings of the current analysis to be integrated with existing literature on implementation of healthy eating and activity interventions within community settings [1, 4–7, 32, 33]. Survey measures assessing a number of constructs from the CFIR were positively related to self-efficacy and intentions to deliver the HEALTH intervention including from the CFIR innovation domain (complexity, evidence strength and quality, and relative advantage), a CFIR inner setting domain (mission alignment), and the RE-AIM adoption and implementation domains. The study did not find a relationship with body mass index or identify differences by most sociodemographic characteristics; however, self-efficacy and implementation intentions were lower among parent educators who identified as white non-Hispanic than among those identifying as another race/ethnicity.

The findings that characteristics of the innovation are correlated with the extent the innovation is taken up in practice are aligned with the findings of other studies in settings serving families with young children. For example, in a study exploring implementation of a healthy eating intervention in early care and education settings, Swindle et al. found positive correlations between perception of innovation, relative advantage and level of institutionalization and fidelity to the WISE (We Inspire Smart Eating) intervention [34]. This suggests the importance of developing interventions to be delivered in community-based settings with the providers who will be asked to deliver them and in a way that supports a fit of the intervention with practice. Involving providers in developing interventions supports a development process where the final intervention has a relative advantage to the current practice and is not perceived as overly complex [35–37]. 

When considering the characteristics of the providers themselves, it is interesting that self-efficacy and implementation intentions were lower among parent educators who identified as white non-Hispanic than among those identifying as another race/ethnicity. This may have implications for HEALTH impact on health equity, given the greater burden of chronic diseases among Americans who identify as Black or African American. Other literature in community settings has also found greater intervention uptake among populations who have been minoritized. When exploring implementation of a healthy eating and activity intervention in churches, Wilcox et al. found differences by race, though at the organizational level (i.e., predominantly African American (versus White) congregations), with greater implementation of the Faith, Activity, and Nutrition intervention at predominantly African churches [5]. Additional research is needed to understand the potential for providers in other community settings, particularly home visiting, to adopt and implement healthy eating and activity interventions, given the diversity of populations they serve and the importance of healthy eating and activity interventions within the home environment.

Further, while it might be expected that body mass index of parent educators is associated with their self-efficacy and/or intent to deliver an intervention, previous work among healthcare providers exploring the barriers to delivering healthy weight content has found mixed results [9, 10]. Similarly, this study did not find a statistically significant association between body mass index of parent educators and their self-efficacy and/or intent to deliver an intervention; further research among community providers should investigate this finding. Particularly in community settings, where discussing behaviors related to health and weight are not part of the typical practice, it may be assumed that providers may be uncomfortable discussing these topics, particularly if their feelings regarding their own weight and weight-related behaviors are complicated; however empirical literature describing this relationship was not available. In the context of home visiting, it is typical for providers to discuss challenging issues related to parenting (e.g., discipline). Parent educators remind the parents that they are not perfect parents themselves, and this type of relationship building may thread through how parent educators are able to adopt and implement a healthy eating and activity intervention. As part of routine practice, parent educators are trained to discuss challenging topics and to leverage the strong relationships they build to support discussing topics they may initially find uncomfortable.

This study is strengthened by its attention to implementation-related factors, as guided by CFIR and RE-AIM, with a community-based home visiting model and inclusion of sites across the United States. The study is limited by the secondary and cross-sectional nature of the design, which prevents an understanding of causality. In addition, the study did not have a mechanism to assess why parent educators did not complete the post-training survey, but participants were assured that participation in each aspect of data collection was voluntary. Further, all measures are self-reported, and the outcomes assessed are the parent educators’ self-efficacy and intentions to deliver the intervention rather than their actual delivery. Future work should explore longitudinal and experimental designs and assess actual intervention delivery to better understand the relationships this analysis identified.

## Conclusions

Community providers have important skills in interacting with people, particularly those who may not be connected with clinical care, yet when implementing healthy eating and activity content within community settings, it is important to consider what factors may be related to provider intent and provider self-efficacy to deliver the content. Specifically, mission alignment, complexity, evidence strength and quality, and relative advantage may be important given providers at community organizations that not designed to deliver health promotion content may have limited experience with such content. Future work is needed to develop strategies to best support providers in community settings with delivering health-related content.

## Data Availability

The datasets used/analyzed during the current study are available from the corresponding author on reasonable request.

## Abbreviations

HEALTH: Healthy Eating and Active Living Taught at Home
PAT: Parents as Teachers
CFIR: Consolidated Framework for Implementation Research
RE-AIM: Reach, Effectiveness, Adoption, Implementation, and Maintenance
SE: Self-efficacy
